# Supplementary material for: Iterative improvement in the automatic modular design of robot swarms
Source: PeerJ Comput Sci. 2020 Dec 7;6:e322. doi: 10.7717/peerj-cs.322 (PMC7924708; doi:10.7717/peerj-cs.322)
Supplement: Supplemental Information 3 [file peerj-cs-06-322-s003.zip › argos3/doc/api/standalone/a00520_source.html]

ARGoS: plugins/robots/generic/simulator/colored\_blob\_perspective\_camera\_default\_sensor.cpp Source File


- Main Page
- Related Pages
- Namespaces
- Classes
- Files

- File List
- File Members

# plugins/robots/generic/simulator/colored\_blob\_perspective\_camera\_default\_sensor.cpp

Go to the documentation of this file.

```
00001 #include "colored_blob_perspective_camera_default_sensor.h"
00002 #include <argos3/core/simulator/simulator.h>
00003 #include <argos3/core/simulator/space/positional_indices/positional_index.h>
00004 #include <argos3/core/simulator/entity/composable_entity.h>
00005 #include <argos3/core/simulator/entity/embodied_entity.h>
00006 #include <argos3/plugins/simulator/entities/led_entity.h>
00007 #include <argos3/plugins/simulator/entities/perspective_camera_equipped_entity.h>
00008 #include <argos3/plugins/simulator/media/led_medium.h>
00009 
00010 namespace argos {
00011 
00012    /****************************************/
00013    /****************************************/
00014 
00015    class CPerspectiveCameraLEDCheckOperation : public CPositionalIndex<CLEDEntity>::COperation {
00016 
00017    public:
00018 
00019       CPerspectiveCameraLEDCheckOperation(
00020          CCI_ColoredBlobPerspectiveCameraSensor::TBlobList& t_blobs,
00021          CPerspectiveCameraEquippedEntity& c_cam_entity,
00022          CEmbodiedEntity& c_embodied_entity,
00023          CControllableEntity& c_controllable_entity,
00024          bool b_show_rays,
00025          Real f_noise_std_dev) :
00026          m_tBlobs(t_blobs),
00027          m_cCamEntity(c_cam_entity),
00028          m_cEmbodiedEntity(c_embodied_entity),
00029          m_cControllableEntity(c_controllable_entity),
00030          m_bShowRays(b_show_rays),
00031          m_fNoiseStdDev(f_noise_std_dev),
00032          m_pcRNG(NULL) {
00033          m_pcRootSensingEntity = &m_cEmbodiedEntity.GetRootEntity();
00034          if(m_fNoiseStdDev > 0.0f) {
00035             m_pcRNG = CRandom::CreateRNG("argos");
00036          }
00037       }
00038       virtual ~CPerspectiveCameraLEDCheckOperation() {
00039          while(! m_tBlobs.empty()) {
00040             delete m_tBlobs.back();
00041             m_tBlobs.pop_back();
00042          }
00043       }
00044 
00045       virtual bool operator()(CLEDEntity& c_led) {
00046          /* Process this LED only if it's lit */
00047          if(c_led.GetColor() != CColor::BLACK) {
00048             /* Filter out the LEDs belonging to the sensing entity by checking if they share the same parent entity */
00049             if(m_pcRootSensingEntity == &c_led.GetRootEntity()) return true;
00050             /* If we are here, it's because the LED must be processed */
00051             /* Set the end of the ray for occlusion checking */
00052             m_cOcclusionCheckRay.SetEnd(c_led.GetPosition());
00053             /* Calculate the vector to LED in the camera-anchor frame of reference */
00054             m_cLEDRelative = c_led.GetPosition();
00055             m_cLEDRelative -= m_cCamEntity.GetAnchor().Position;
00056             m_cLEDRelative.Rotate(m_cInvCameraOrient);
00057             /* Calculate the projection of the LED vector into the camera direction */
00058             Real fDotProd = m_cLEDRelative.GetX();
00059             /* The blob is visible if
00060              * 1. It is within the distance range AND
00061              * 2. It is within the aperture range AND
00062              * 3. There are no occlusions
00063              */
00064             if(fDotProd < m_cCamEntity.GetRange() &&
00065                ACos(fDotProd / m_cLEDRelative.Length()) < m_cCamEntity.GetAperture() &&
00066                !GetClosestEmbodiedEntityIntersectedByRay(m_sIntersectionItem,
00067                                                          m_cOcclusionCheckRay,
00068                                                          m_cEmbodiedEntity)) {
00069                /* The LED is visibile */
00070                /* Calculate the intersection point between the LED ray and the image plane */
00071                m_cLEDRelative.Normalize();
00072                m_cLEDRelative *= m_cCamEntity.GetFocalLength() / m_cLEDRelative.GetX();
00073                /*
00074                 * The image plane is perpendicular to the local X axis
00075                 * Y points to the left, Z up, the origin is in the image center
00076                 * To find the pixel (i,j), we need to flip both Y and Z, and translate the origin
00077                 * So that the origin is up-left, the i axis goes to the right, and the j axis goes down
00078                 */
00079                SInt32 unI =
00080                   - m_cCamEntity.GetImagePxWidth() /
00081                   m_cCamEntity.GetImageMtWidth() *
00082                   (m_cLEDRelative.GetY() -
00083                    m_cCamEntity.GetImageMtWidth() * 0.5f);
00084                SInt32 unJ =
00085                   - m_cCamEntity.GetImagePxHeight() /
00086                   m_cCamEntity.GetImageMtHeight() *
00087                   (m_cLEDRelative.GetZ() -
00088                    m_cCamEntity.GetImageMtHeight() * 0.5f);
00089                /* Make sure (i,j) is within the limits */
00090                if((unI >= m_cCamEntity.GetImagePxWidth() || unI < 0) ||
00091                   (unJ >= m_cCamEntity.GetImagePxHeight() || unJ < 0))
00092                   return true;
00093                /* Add new blob */
00094                m_tBlobs.push_back(
00095                   new CCI_ColoredBlobPerspectiveCameraSensor::SBlob(
00096                      c_led.GetColor(), unI, unJ));
00097                /* Draw ray */
00098                if(m_bShowRays) {
00099                   m_cControllableEntity.AddCheckedRay(
00100                      false,
00101                      CRay3(m_cCamEntity.GetAnchor().Position,
00102                            c_led.GetPosition()));
00103                }
00104             }
00105          }
00106          return true;
00107       }
00108       
00109       void Setup() {
00110          /* Erase blobs */
00111          while(! m_tBlobs.empty()) {
00112             delete m_tBlobs.back();
00113             m_tBlobs.pop_back();
00114          }
00115          /* Reset ray start */
00116          m_cOcclusionCheckRay.SetStart(m_cCamEntity.GetAnchor().Position);
00117          /* Calculate inverse of camera orientation */
00118          m_cInvCameraOrient = m_cCamEntity.GetAnchor().Orientation.Inverse();
00119       }
00120       
00121    private:
00122       
00123       CCI_ColoredBlobPerspectiveCameraSensor::TBlobList& m_tBlobs;
00124       CPerspectiveCameraEquippedEntity& m_cCamEntity;
00125       CEmbodiedEntity& m_cEmbodiedEntity;
00126       CControllableEntity& m_cControllableEntity;
00127       CQuaternion m_cInvCameraOrient;
00128       bool m_bShowRays;
00129       CEntity* m_pcRootSensingEntity;
00130       CRadians m_cTmp1, m_cTmp2;
00131       CVector3 m_cLEDRelative;
00132       SEmbodiedEntityIntersectionItem m_sIntersectionItem;
00133       CRay3 m_cOcclusionCheckRay;
00134       Real m_fNoiseStdDev;
00135       CRandom::CRNG* m_pcRNG;
00136    };
00137 
00138    /****************************************/
00139    /****************************************/
00140 
00141    CColoredBlobPerspectiveCameraDefaultSensor::CColoredBlobPerspectiveCameraDefaultSensor() :
00142       m_bEnabled(false),
00143       m_pcCamEntity(NULL),
00144       m_pcControllableEntity(NULL),
00145       m_pcEmbodiedEntity(NULL),
00146       m_pcLEDIndex(NULL),
00147       m_pcEmbodiedIndex(NULL),
00148       m_bShowRays(false) {
00149    }
00150 
00151    /****************************************/
00152    /****************************************/
00153 
00154    CColoredBlobPerspectiveCameraDefaultSensor::~CColoredBlobPerspectiveCameraDefaultSensor() {
00155    }
00156 
00157    /****************************************/
00158    /****************************************/
00159 
00160    void CColoredBlobPerspectiveCameraDefaultSensor::SetRobot(CComposableEntity& c_entity) {
00161       /* Get omndirectional camera equipped entity */
00162       m_pcCamEntity = &(c_entity.GetComponent<CPerspectiveCameraEquippedEntity>("perspective_camera"));
00163       /* Get controllable entity */
00164       m_pcControllableEntity = &(c_entity.GetComponent<CControllableEntity>("controller"));
00165       /* Get embodied entity */
00166       m_pcEmbodiedEntity = &(c_entity.GetComponent<CEmbodiedEntity>("body"));
00167    }
00168 
00169    /****************************************/
00170    /****************************************/
00171 
00172    void CColoredBlobPerspectiveCameraDefaultSensor::Init(TConfigurationNode& t_tree) {
00173       try {
00174          /* Parent class init */
00175          CCI_ColoredBlobPerspectiveCameraSensor::Init(t_tree);
00176          /* Show rays? */
00177          GetNodeAttributeOrDefault(t_tree, "show_rays", m_bShowRays, m_bShowRays);
00178          /* Parse noise */
00179          Real fNoiseStdDev = 0.0f;
00180          GetNodeAttributeOrDefault(t_tree, "noise_std_dev", fNoiseStdDev, fNoiseStdDev);
00181          /* Get LED medium from id specified in the XML */
00182          std::string strMedium;
00183          GetNodeAttribute(t_tree, "medium", strMedium);
00184          m_pcLEDIndex = &(CSimulator::GetInstance().GetMedium<CLEDMedium>(strMedium).GetIndex());
00185          /* Create check operation */
00186          m_pcOperation = new CPerspectiveCameraLEDCheckOperation(
00187             m_sReadings.BlobList,
00188             *m_pcCamEntity,
00189             *m_pcEmbodiedEntity,
00190             *m_pcControllableEntity,
00191             m_bShowRays,
00192             fNoiseStdDev);
00193       }
00194       catch(CARGoSException& ex) {
00195          THROW_ARGOSEXCEPTION_NESTED("Error initializing the colored blob perspective camera default sensor", ex);
00196       }
00197    }
00198 
00199    /****************************************/
00200    /****************************************/
00201 
00202    void CColoredBlobPerspectiveCameraDefaultSensor::Update() {
00203       if(m_bEnabled) {
00204          /* Increase data counter */
00205          ++m_sReadings.Counter;
00206          /* Prepare the operation */
00207          m_pcOperation->Setup();
00208          /* Calculate the sensing box */
00209          Real fHalfRange  = m_pcCamEntity->GetRange() * 0.5f;
00210          Real fHalfSide   = fHalfRange * Tan(m_pcCamEntity->GetAperture());
00211          /* Box center */
00212          CVector3 cCenter(fHalfRange, 0.0f, 0.0f);
00213          cCenter.Rotate(m_pcCamEntity->GetAnchor().Orientation);
00214          cCenter += m_pcCamEntity->GetAnchor().Position;
00215          /* Box half size */
00216          CVector3 cCorner(fHalfRange, fHalfSide, fHalfSide);
00217          cCorner.Rotate(m_pcCamEntity->GetAnchor().Orientation);
00218          CVector3 cHalfSize(
00219             Abs(cCorner.GetX()),
00220             Abs(cCorner.GetY()),
00221             Abs(cCorner.GetZ()));
00222          /* Go through LED entities in box range */
00223          m_pcLEDIndex->ForEntitiesInBoxRange(
00224             cCenter, cHalfSize, *m_pcOperation);
00225       }
00226    }
00227 
00228    /****************************************/
00229    /****************************************/
00230 
00231    void CColoredBlobPerspectiveCameraDefaultSensor::Reset() {
00232       m_sReadings.Counter = 0;
00233       m_sReadings.BlobList.clear();
00234    }
00235 
00236    /****************************************/
00237    /****************************************/
00238 
00239    void CColoredBlobPerspectiveCameraDefaultSensor::Destroy() {
00240       delete m_pcOperation;
00241    }
00242 
00243    /****************************************/
00244    /****************************************/
00245 
00246    void CColoredBlobPerspectiveCameraDefaultSensor::Enable() {
00247       m_pcCamEntity->Enable();
00248       m_bEnabled = true;
00249    }
00250 
00251    /****************************************/
00252    /****************************************/
00253 
00254    void CColoredBlobPerspectiveCameraDefaultSensor::Disable() {
00255       m_pcCamEntity->Disable();
00256       m_bEnabled = false;
00257    }
00258 
00259    /****************************************/
00260    /****************************************/
00261 
00262    REGISTER_SENSOR(CColoredBlobPerspectiveCameraDefaultSensor,
00263                    "colored_blob_perspective_camera", "default",
00264                    "Carlo Pinciroli [ilpincy@gmail.com]",
00265                    "1.0",
00266                    "A generic perspective camera sensor to detect colored blobs.",
00267                    "This sensor accesses an perspective camera that detects colored blobs. The\n"
00268                    "sensor returns a list of blobs, each defined by a color and a position with\n"
00269                    "respect to the robot reference point on the ground. In controllers, you must\n"
00270                    "include the ci_colored_blob_perspective_camera_sensor.h header.\n\n"
00271                    "REQUIRED XML CONFIGURATION\n\n"
00272                    "  <controllers>\n"
00273                    "    ...\n"
00274                    "    <my_controller ...>\n"
00275                    "      ...\n"
00276                    "      <sensors>\n"
00277                    "        ...\n"
00278                    "        <colored_blob_perspective_camera implementation=\"default\"\n"
00279                    "                                         medium=\"leds\" />\n"
00280                    "        ...\n"
00281                    "      </sensors>\n"
00282                    "      ...\n"
00283                    "    </my_controller>\n"
00284                    "    ...\n"
00285                    "  </controllers>\n\n"
00286                    "The 'medium' attribute must be set to the id of the leds medium declared in the\n"
00287                    "<media> section.\n\n"
00288                    "OPTIONAL XML CONFIGURATION\n\n"
00289                    "It is possible to draw the rays shot by the camera sensor in the OpenGL\n"
00290                    "visualization. This can be useful for sensor debugging but also to understand\n"
00291                    "what's wrong in your controller. In OpenGL, the rays are drawn in cyan when\n"
00292                    "they are not obstructed and in purple when they are. In case a ray is\n"
00293                    "obstructed, a black dot is drawn where the intersection occurred.\n"
00294                    "To turn this functionality on, add the attribute \"show_rays\" as in this\n"
00295                    "example:\n\n"
00296                    "  <controllers>\n"
00297                    "    ...\n"
00298                    "    <my_controller ...>\n"
00299                    "      ...\n"
00300                    "      <sensors>\n"
00301                    "        ...\n"
00302                    "        <colored_blob_perspective_camera implementation=\"default\"\n"
00303                    "                                         medium=\"leds\" />\n"
00304                    "                                         show_rays=\"true\" />\n"
00305                    "        ...\n"
00306                    "      </sensors>\n"
00307                    "      ...\n"
00308                    "    </my_controller>\n"
00309                    "    ...\n"
00310                    "  </controllers>\n\n"
00311                    "It is possible to add uniform noise to the blobs, thus matching the\n"
00312                    "characteristics of a real robot better. This can be done with the attribute\n"
00313                    "\"noise_std_dev\".\n\n"
00314                    "  <controllers>\n"
00315                    "    ...\n"
00316                    "    <my_controller ...>\n"
00317                    "      ...\n"
00318                    "      <sensors>\n"
00319                    "        ...\n"
00320                    "        <colored_blob_perspective_camera implementation=\"default\"\n"
00321                    "                                         medium=\"leds\" />\n"
00322                    "                                         noise_std_dev=\"0.1\" />\n"
00323                    "        ...\n"
00324                    "      </sensors>\n"
00325                    "      ...\n"
00326                    "    </my_controller>\n"
00327                    "    ...\n"
00328                    "  </controllers>\n",
00329                    "Usable"
00330       );
00331 
00332 }
```

---

Generated on 10 Jul 2018 for ARGoS by 
 1.6.1
